# SnapShot Freeze 2.0, a second-generation motion-correction algorithm, improves mitral valve image quality in preoperative cardiac computed tomography for mitral regurgitation

**DOI:** 10.1007/s11604-025-01940-0

**Published:** 2026-01-17

**Authors:** Hiromi Hashimura, Kentaro Nishiuchi, Yu Izawa, Kei Honde, Kazuki Ishikawa, Hiroaki Takahashi, Takamichi Murakami

**Affiliations:** 1 Department of Radiology, Kobe University Hospital, Kobe University Graduate School of Medicine, 7-5-2, Kusunoki-cho, Chuo-ku, Kobe, Hyogo 650−0017 Japan; 2 Division of Cardiovascular Medicine, Department of Internal Medicine, Kobe University Graduate School of Medicine, Kobe, Japan; 3 Center for Radiology and Radiation Oncology, Kobe University Hospital, Kobe, Japan; 4 Department of Cardiovascular Surgery, Kobe University Graduate School of Medicine, Kobe, Japan

**Keywords:** Computed tomography angiography, Mitral regurgitation, Mitral valve, Mitral valve plasty, Motion-correction algorithm

## Abstract

**Background:**

Preoperative cardiac CT for mitral valve assessment is crucial, and improvement of image quality using motion-correction algorithms remains unreported.

**Materials and methods:**

We retrospectively analyzed 141 datasets from 47 patients with mitral regurgitation, reconstructing images acquired using a CT scanner with 16-cm z-axis coverage with prospective electrocardiography gating utilizing standard (STD), SnapShot Freeze 1.0 (SSF), and SnapShot Freeze 2.0 (SSF2) algorithms. Two radiologists blinded to reconstruction methods, cardiac cycle, and patient information evaluated image quality for mitral valve leaflets (A1-P3) using a five-point Likert scale, assessing 20 cardiac phases for the A2/P2 and systolic phases for all leaflets.

**Results:**

Image quality scored significantly higher using SSF2 compared to STD or SSF reconstruction for all 20 cardiac phases of the A2/P2 and the systolic phase of all leaflets (*P* < 0.05). STD and SSF scores did not differ significantly but were higher in atrial fibrillation than sinus rhythm, a difference not observed with SSF2. No reconstruction method showed significant correlation between heart rate and image quality. The mean systolic phase contributing most to mitral regurgitation diagnosis was 27.7% of the R-R interval. Interobserver agreement was excellent (weighted kappa = 0.956 for A2/P2; 0.915 for A1/P1, A2/P2, and A3/P3).

**Conclusions:**

Mitral valve image quality in preoperative cardiac CT for mitral regurgitation improved significantly using SSF2 reconstruction compared to STD and SSF. Reflected across different cardiac phases and influenced by neither heart rhythm nor rate, findings suggest the robustness of SSF2 for use in various clinical scenarios and to aid surgical planning.

## Introduction

The prevalence of valvular heart disease increases significantly with age, rising from 0.3% in individuals aged 18 to 44 to 11.7% in those 75 and older [1], with more prevalent disease of the mitral valve (MV) than aortic valve [1]. Within mitral valve disease, cases of mitral stenosis, primarily resulting as a consequence of early rheumatic fever, have been dramatically reduced over time, even among the elderly, in response to improved hygiene. Conversely, as the population ages, mitral regurgitation is on the rise [2] and associated with significant morbidity and mortality [3].

The primary treatment for symptomatic severe mitral regurgitation is mitral valve surgery [4], with repair favored over replacement, yielding generally superior outcomes and reduced complications [5]. Accurate morphological and functional characterization of the mitral valve is crucial for preoperative planning in both traditional and emerging minimally invasive procedures, such as transcatheter edge-to-edge repair and transcatheter mitral valve replacement [6–8]. The mitral valve is a complex structure comprising the annulus, leaflets, chordae tendineae, and subvalvular apparatus including papillary muscles. Comprehensive assessment of the entire mitral valve is challenging using two-dimensional transesophageal echocardiography (TEE), and even three-dimensional TEE has limitations due to its narrow field of view. In contrast, computed tomography (CT) allows anatomical evaluation encompassing the left ventricle and left atrium.

Cardiac CT scans are highly susceptible to the effects of heart rate, arrhythmias, and the heart’s own motion, which often lead to artifacts in the images. Motion-correction algorithms have been developed to address this issue and are particularly crucial for patients with elevated heart rates or arrhythmias. The newly released second-generation motion-correction algorithm SnapShot Freeze 2.0 (SSF2, GE HealthCare) succeeds the first-generation algorithm (SnapShot Freeze 1.0, SSF) and significantly improves motion correction for coronary arteries and other cardiac structures [9]. SSF2 has been shown to enhance depiction of the aortic valve and coronary arteries in candidates for transcatheter aortic valve implantation (TAVI) [9, 10] and patients with mechanical aortic, mitral, and tricuspid valves [11] and to improve delineation of cardiac structures in pediatric patients with high heart rates [12] and coronary arteries in patients with increased heart rates [13]. We undertook this study to evaluate the previously unexplored ability of SSF2 to improve the quality of mitral valve images in preoperative patients with mitral regurgitation.

## Materials and methods

Our local ethics committee approved this retrospective study, and acquisition of written informed consent for the retrospective analysis was waived in accordance with the Declaration of Helsinki.

### Study population

We identified consecutive patients who underwent preoperative cardiac CT examination for mitral regurgitation between 01 April 2022 and 30 April 2024 from the radiology database of our university hospital. At our institution, patients eligible for mitral valve assessment were those without severe renal impairment, except patients undergoing dialysis, and those who had CT data loss due to sudden arrhythmia during the examination. Prior to scan initiation, when their systolic blood pressure was 90 mmHg or higher, patients were administered nitroglycerin tablets sublingually to dilate the coronary arteries and facilitate assessment. No patient received beta-blockers during the CT scanning.

### Computed tomography protocol

All CT examinations were performed on a scanner with 16-cm (256 × 0.625 mm) z-axis coverage (Revolution™ Apex, GE HealthCare) with patients in supine position and feet towards the gantry. For screening and to check cardiac position, an initial non-contrast, non-gated helical CT scan was obtained with 120-kV tube voltage, gantry rotation speed of 0.28 s/rotation, and scan range from the thorax to pelvis. To acquire data of all cardiac phases after contrast injection, coronary CT angiography (CCTA) was then obtained for one (120% of the R-peak to R-peak [R-R] interval) to three heart rates in non-helical scanning mode with prospective electrocardiography (ECG) gating. Optimal tube voltage and tube current were selected using the automatic exposure control function (Auto Prescription, GE HealthCare) under the following conditions: tube voltage, 80 kVp; noise index, 24; and pre-scan adaptive statistical iterative reconstruction V (pre-ASiR-V, GE HealthCare), 70%. To evaluate the aorta and its main branches, non-gated helical CTA from the thorax to pelvis was performed after a delay of 5.1 s following CCTA. Finally, to assess the veins and abdominal organs, contrast-enhanced CT of the equilibrium phase was performed 120 s after injection of the contrast medium. The contrast agent was administered at a dose of 28 mgI/kg/s for 12 s followed by a 15-second injection of its 40% dilution at the same rate. The bolus tracking technique (SmartPrep, GE HealthCare) was employed to automatically trigger CCTA with the threshold set at 200 HU. The region of interest (ROI) for monitoring the contrast agent was placed in the ascending aorta, and CCTA started 7.4 s after triggering.

### Data construction (Fig. 1)

**Fig. 1:**
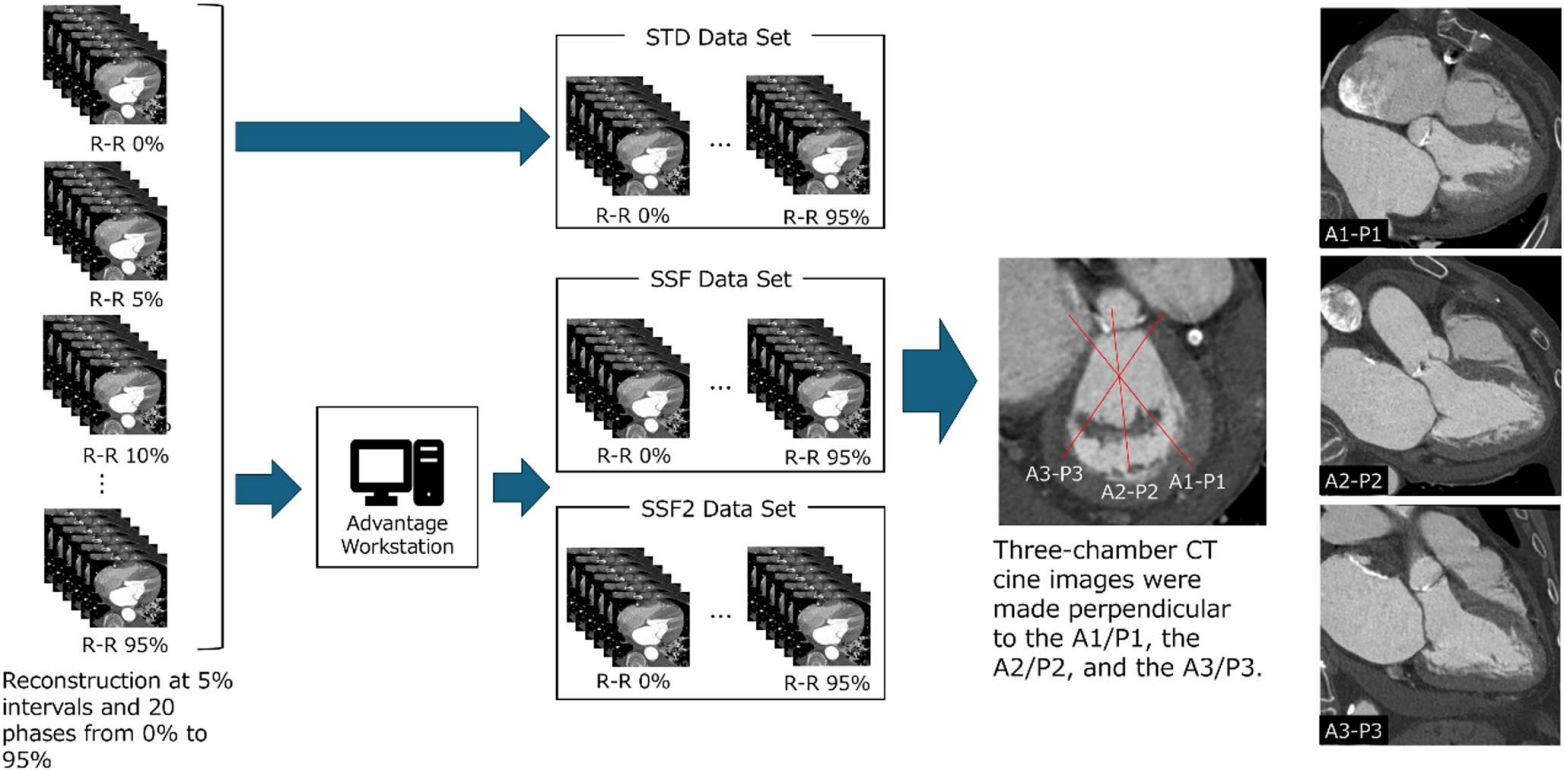
Method of image reconstruction required for analysis after scanning. A1, lateral portion of the anterior mitral leaflet; A2, middle portion of the anterior mitral leaflet; A3, medial portion of the anterior mitral leaflet; CT, computed tomography; P1, lateral scallop of the posterior mitral leaflet; P2, middle scallop of the posterior mitral leaflet; P3, medial scallop of the posterior mitral leaflet; R-R, R-peak to R-peak; SSF, SnapShot Freeze 1.0; SSF2; SnapShot Freeze 2.0. STD, Standard

To evaluate the coronary artery and mitral valve, we reconstructed data acquired by CCTA at 5% R-R intervals between the ECG R-R waves and for 20 phases ranging from the zero to 95% R-R cardiac phases with 240 × 240-mm field of view, 512 × 512 reconstruction matrix, and 0.625-mm reconstruction slice thickness and slice intervals. We reconstructed all images of the 20 phases and images of all 20 cardiac phases utilizing the two motion-correction algorithms‒SnapShot Freeze (SSF, GE HealthCare) and SnapShot Freeze 2 (SSF2, GE HealthCare)‒and standard image reconstruction without motion correction (STD). A standard kernel was used for each of the 20 cardiac phases with the three reconstructions. In all reconstructions, deep learning imaging reconstruction (TrueFidelity, GE HealthCare) was set to high to reduce noise in all images. For subjective evaluation of mitral valve image quality, three-chamber CT cine images were made perpendicular to the A1 (lateral portion of the anterior mitral leaflet)/P1 (lateral scallop of the posterior mitral leaflet), A2 (middle portion of the anterior mitral leaflet)/P2 (middle scallop of the posterior mitral leaflet), and A3 (medial portion of the anterior mitral leaflet)/P3 (medial scallop of the posterior mitral leaflet) from the short-axis view of the left ventricle using all 20 phase images with three types of reconstruction (total 180 images; three types of CT images for 20 phases for three types of reconstruction).

### Image analysis

**Fig. 2:**
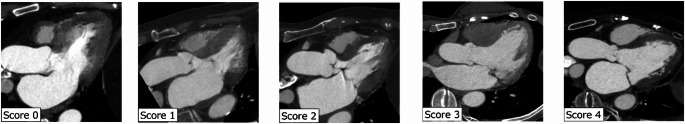
Examples of computed tomography images of the mitral valve with associated radiologist assessment of image quality; both the anterior and posterior leaflets show the same score. Score 0, nondiagnostic (mitral leaflet cannot be identified); Score 1, poor (less than half of the mitral leaflet is visible); Score 2, adequate (a portion of the mitral leaflet is not depicted); Score 3, good (depiction of the entire leaflet with part of the leaflet blurred); Score 4, excellent (entire leaflet is well defined with no blurring)

**Fig. 3:**
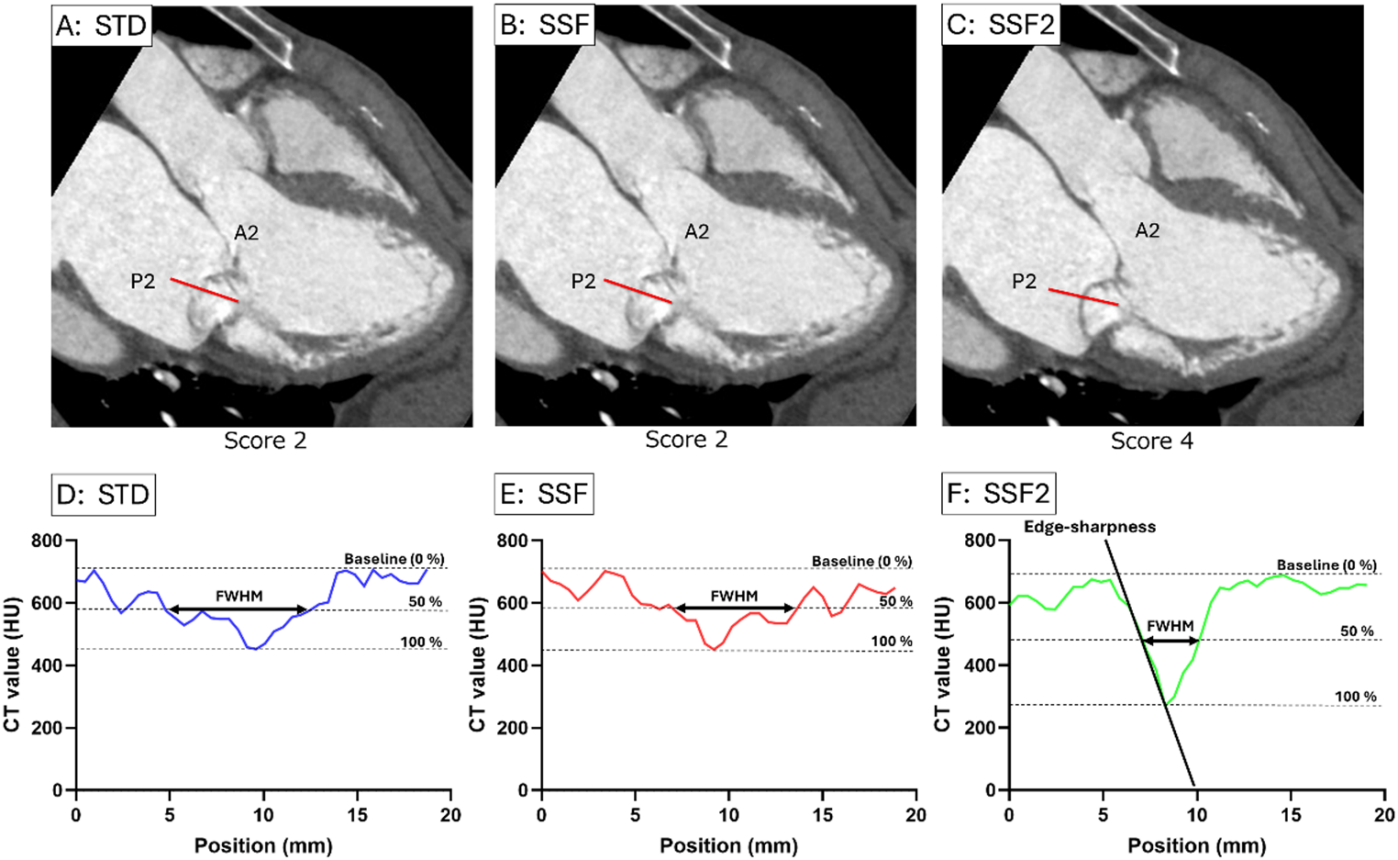
Cardiac computed tomography angiography of a 71-year-old male patient (height, 164 cm; weight, 47 kg; body mass index, 17.4 kg/m²; heart rate, 50 bpm) with mitral valve prolapse of the P3 segment, complicated by atrial fibrillation. The CT scan was performed with the patient’s heart rate of 50 bpm (CTDIvol, 22.8 mGy; DLP, 364.4 mGy·cm). Quantitative image quality assessment: the full width at half maximum (FWHM) values were 7.7 mm for STD, 6.3 mm for SSF, and 2.4 mm for SSF2. The edge sharpness obtained with SSF2 was measured as -141.4 [HU/mm]. Measurement of this parameter was challenging for the STD and SSF reconstructions because their corresponding CT profile curves exhibited multi-peaked (multimodal) curves. **A** Standard image of the A2/P2 segment (Score 2). **B** SSF image of the A2/P2 segment (Score 2). **C** SSF2 image of the A2/P2 segment (Score 4). **D** CT profile curve of STD image of the A2/P2 segment. **E** CT profile curve of SSF image of the A2/P2 segment. **F** CT profile curve of SSF2 image of the A2/P2 segment. A2, middle portion of the anterior mitral leaflet; P2, middle scallop of the posterior mitral leaflet; P3, medial scallop of the posterior mitral leaflet; SSF, SnapShot Freeze 1.0; SSF2, SnapShot Freeze 2.0

Two radiologists, HH, with 18 years of experience in cardiac CT analysis and interpretation, and KN, with four years of experience, who were blinded to clinical information, visually assessed subjective image quality for all 20 cardiac phases for the A2/P2 and for only the systolic-phase images that contributed most to the diagnosis of mitral valve regurgitation for the A1/P1 and A3/P3 for each of the three patterns‒two with motion correction (SSF and SSF2) and one without (STD). Blinded to reconstruction methods, cardiac cycle, and patient information, the two reviewers performed qualitative assessments of subjective image quality in each dataset in random order for all cardiac phases with different construction methods. They rated image quality from zero to four based on a five-point Likert scale according to the degree of blurring from artifact distortion and scored each acquisition independently from the other. Image quality of the mitral leaflet was scored as excellent when the entire leaflet was well defined without blurring (Score 4); good when part of the leaflet was blurred (Score 3); adequate when only a portion of the leaflet was depicted (Score 2); poor when less than half of the leaflet was visible (Score 1); or nondiagnostic when the mitral leaflet could not be identified (Score 0) (Fig. 2). This visual assessment was conducted by mindful consideration of key quantitative image quality metrics‒contrast difference(ΔHU); valve thickness/sharpness (full width at half medium [FWHM]); signal-to-noise ratio (SNR); and edge sharpness (ΔHU/Δx, maximum gradient) (Fig. 3).

Disagreement between the two reviewers’ ratings of image quality was resolved by averaging their scores to provide a more comprehensive assessment.

### Radiation dose

To calculate the radiation dose exposure of CCTA, the CT dose index (CTDIvol, mGy) and CT dose-length product (DLP, mGy*cm) were recorded for all scans. The effective dose (ED) was calculated as the product of the DLP and a conversion coefficient (K) for the chest (K = 0.014 mSv/mGy*cm).

### Statistical analysis

We performed all statistical analyses with GraphPad Prism version 10.3.1 (San Diego, California, USA), using Fleiss’ kappa to assess interobserver agreement among the image quality scores of mitral valves; the Shapiro-Wilk test to test normality because the number of cases was small; and the Wilcoxon test to compare the differences in image quality scores between STD and SSF, STD and SSF2, and SSF and SSF2 for the 20 phases of the A2 and P2. We used the Wilcoxon matched-pairs signed rank test to analyze the systolic-phase images that contributed most to the diagnosis of mitral regurgitation for the A1, A2, A3, P1, P2, and P3, and we employed the Kruskal-Wallis test to analyze the statistical relationship between the systolic phase of the heart and the heart rate at the time of scanning. We utilized the Kolmogorov-Smirnov test to compare atrial fibrillation (AF) and sinus rhythm (SR) and analyze systolic image scores in three different image sets (STD, SSF, and SSF2) and used linear regression to examine the correlation between heart rate at the time of imaging and the score for each reconstruction method. The Kruskal-Wallis test was used to evaluate the difference in score increase due to heart rate.

## Results

### Patient characteristics

We analyzed 141 datasets of 47 cases in which patients were diagnosed with severe mitral regurgitation using transthoracic echocardiography between 01 April 2022 and 30 April 2024 and then underwent CT for detailed evaluation prior to mitral valve annuloplasty. Table 1 summarizes the clinical characteristics and CT scanning parameters. Thirty of the 47 patients (63.8%) were male. The patients’ mean age was 65.3 years; mean weight, 60.4 kg; and mean body mass index (BMI), 25.27 kg/m^2^. The ED of CCTA and the non-gated helical CTA was 4.58 ± 2.59 mSv. Eight of the 47 cases (17%) of mitral regurgitation were functional, and 39 (83%) were degenerative.

**Table 1:** Characteristics of 47 patients diagnosed with severe mitral regurgitation who underwent preoperative cardiac computed tomography examination for mitral regurgitation

Demographic characteristics (Number of patients = 47)	Results
Mean age (years)	65.3 ± 13.0
Number of men	30 (63.8%)
Number of women	17 (36.2%)
Weight (kg)	60.4 ± 14.7
Height (cm)	158.9 ± 17.7
Body mass index (kg/m^2^)	25.3 ± 14.3
*Clinical parameters*	
Hypertension	16 (34.0%)
Diabetes mellitus	5 (10.6%)
Dyslipidemia	8 (17.0%)
Coronary artery disease	3 (6.4%)
*Blood test results*	
B-type natriuretic peptide (pg/mL)	336 (3-6519)
eGFR (mL/min/1.73 m^2^)	63 ± 22
*Echocardiography findings*	
LVDd (mm)	51.4 ± 7.0
LVDs (mm)	32.5 ± 6.8
IVSd (mm)	9.8 ± 2.8
PWd (mm)	10.6 ± 2.6
LVEF (%)	61.2 ± 9.3
LAV	127.2 ± 54.8
LAVI	79.8 ± 43.2
MRV	98.3 ± 45.0
MRF	61.2 ± 15.5
*CCTA information*	
Heart rate before and after scan (beats per minute)	
minimum	57.1 ± 15.6
maximum	88.26 ± 26.7
average	70.0 ± 16.0
Heart rate during scan (beats/min)	65.8 ± 15.6
atrial fibrillation	16 (34.0%)
best systolic phase (%)	27.7
CTDIvol (mGy)	20.5 ± 11.9
DLP (mGy*cm)	327.2 ± 185.1
ED (mSv)	4.58 ± 2.59

Data are mean ± standard deviation or number (percentage). CTDIvol, computed tomography dose index volume; DLP, dose length product; ED, effective dose; eGFR, estimated glomerular filtration rate; HR, heart rate; IVSd, interventricular septum diameter; LAV, left atrial volume; LAVI, left atrial volume index; LVDd, left ventricular diastolic diameter; LVDs, left ventricular systolic diameter; LVEF, left ventricular ejection fraction; MRF, mitral regurgitation fraction; MRV, mitral regurgitation volume; PWd, posterior wall diameter

### Interobserver agreement

Interobserver agreement was almost perfect for all images of the 20 cardiac phases for the A2/P2, with weighted kappa values of 0.957 (95% CI 0.925, 0.951) for STD, 0.936 (95% CI 0.923, 0.950) for SSF, and 0.957 (95% CI 0.923, 0.949) for SSF2. As well, inter-observer agreement for the diagnosis of mitral regurgitation at each mitral valve scallop (A1, A2, A3, P1, P2, and P3) was almost perfect, based on the systolic images that contributed most to the diagnosis. The weighted kappa values were 0.958 (95% CI 0.907, 0.976) for STD, 0.958 (95% CI 0.907, 0.975) for SSF, and 0.927 (95% CI 0.872, 0.961) for SSF2. Calculation of the weighted kappa assumed that the categories were ordered, considered the distance between the two raters, and utilized linear weights.

### The best systolic phases of images

**Fig. 4:**
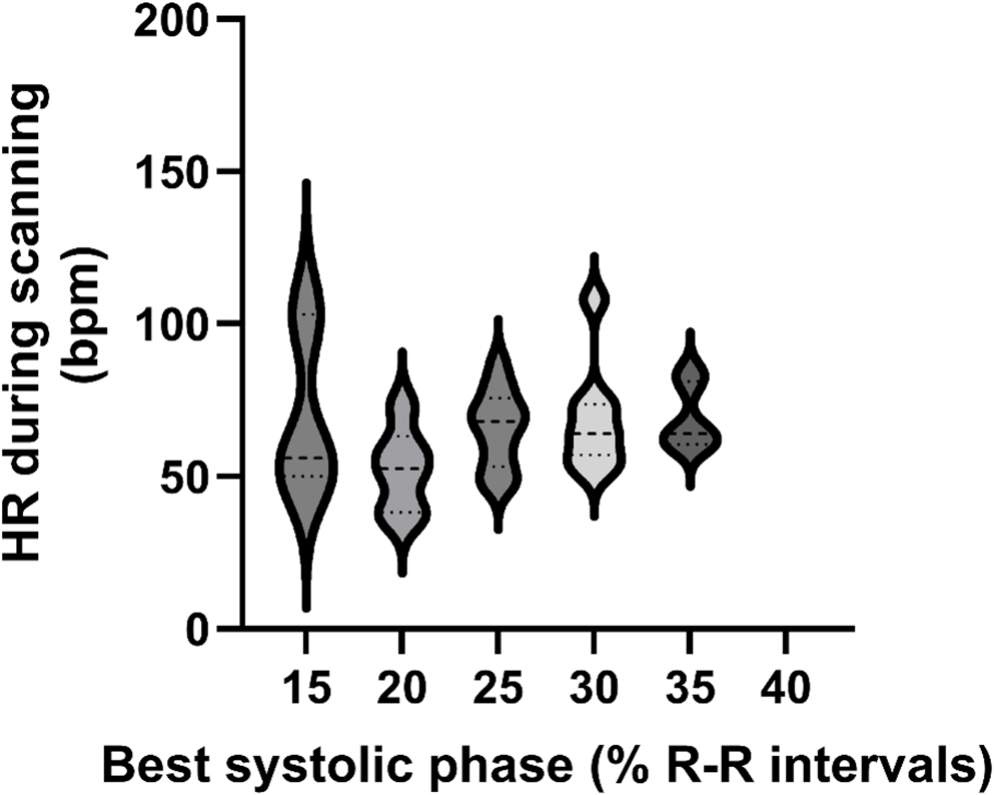
Violin plots showing the patient’s heart rate (HR) at the time of scanning during the best systolic phases. bpm, beats per minute; HR, heart rate; R-R, R-peak-to-R-peak

The mean systolic phase that contributed most to the diagnosis of MV regurgitation was 27.7% (minimum 15%, maximum 40%) of the R-R intervals on electrocardiography. During the scan, heart rate did not differ significantly among the best systolic phases of the images (*P* = 0.33) (Fig. 4).

### Image quality

- Subjective image quality scores for all 20 cardiac phases for the A2/P2.

The mean subjective image quality scores across all 20 cardiac phases were significantly higher for SSF2 compared to STD and SSF. Specifically, the scores for the A2 scallop were 0.75 ± 0.87 for STD, 2.75 ± 0.87 for SSF, and 2.92 ± 0.90 for SSF2. For the P2 scallop, the scores were 2.50 ± 1.03 for STD, 2.50 ± 1.03 for SSF, and 2.7 ± 1.06 for SSF2. STD and SSF scores did not differ significantly for either the A2 (*P* = 0.75) or P2 (*P* = 0.91).

Table 2 shows the image scores for the A2 and P2 in each cardiac phase for images obtained using the STD, SSF, and SSF2 methods. With the exception of the 20% R-R-phase P2 images, the SSF2 scores were significantly higher than those of STD and SSF in the systolic phase from the five to 40% R-R intervals.

**Table 2:** Image scores for the A2 and P2 in each cardiac phase for images obtained using the STD, SSF, and SSF2 reconstruction methods

A2				*P*			P2				*P*		
	STD	SSF	SSF2	STD vs. SSF	STD vs. SSF2	SSF vs. SSF2		STD	SSF	SSF2	STD vs. SSF	STD vs. SSF2	SSF vs. SSF2
0%	2.46 ± 0.78	2.44 ± 0.78	2.52 ± 0.86	> 0.999	0.375	0.313	0%	2.05 ± 0.87	2.10 ± 0.86	0.86 ± 0.94	> 0.999	0.109	0.125
5%	2.55 ± 0.85	2.53 ± 0.82	2.71 ± 0.82	> 0.999	**0.035**	**0.020**	5%	2.26 ± 0.87	2.27 ± 0.85	2.44 ± 0.89	> 0.999	**0.044**	**0.048**
10%	2.75 ± 0.87	2.76 ± 0.86	3.05 ± 0.92	> 0.999	**0.001**	**0.001**	10%	2.37 ± 0.87	2.40 ± 0.87	2.77 ± 0.94	0.500	**< 0.001**	**0.002**
15%	2.90 ± 0.79	2.91 ± 0.78	3.17 ± 0.79	> 0.999	**0.011**	**0.011**	15%	2.64 ± 0.82	2.67 ± 0.86	2.92 ± 0.88	0.625	**0.003**	**0.007**
20%	2.72 ± 0.94	2.71 ± 0.91	3.13 ± 0.82	> 0.999	**0.002**	**0.002**	20%	2.71 ± 0.91	2.71 ± 0.86	2.89 ± 0.86	> 0.999	0.064	0.064
25%	2.86 ± 0.80	2.84 ± 0.81	3.23 ± 0.81	> 0.999	**0.001**	**0.000**	25%	2.54 ± 1.07	2.51 ± 1.06	2.95 ± 0.99	0.500	**0.001**	**< 0.001**
30%	2.84 ± 0.94	2.84 ± 0.94	3.35 ± 0.79	> 0.999	**< 0.001**	**< 0.001**	30%	2.66 ± 1.04	2.67 ± 1.02	3.10 ± 0.86	> 0.999	**0.001**	**0.001**
35%	2.82 ± 0.82	2.78 ± 0.81	3.07 ± 0.90	0.500	**0.003**	**0.001**	35%	2.57 ± 1.06	2.60 ± 1.06	2.93 ± 1.00	> 0.999	**< 0.001**	**0.002**
40%	2.44 ± 0.93	2.46 ± 0.91	2.72 ± 0.95	0.750	**0.007**	**0.005**	40%	2.32 ± 1.11	2.36 ± 1.06	2.57 ± 1.10	0.625	**0.010**	**0.020**
45%	2.28 ± 0.93	2.21 ± 0.91	2.32 ± 0.99	0.500	0.795	0.227	45%	1.97 ± 1.08	1.99 ± 1.09	2.14 ± 1.17	> 0.999	0.152	0.210
50%	2.56 ± 0.92	2.59 ± 0.92	2.69 ± 0.98	> 0.999	0.119	0.145	50%	2.10 ± 1.04	2.06 ± 1.03	2.17 ± 1.11	0.500	0.344	0.168
55%	2.57 ± 0.93	2.60 ± 0.95	2.57 ± 1.00	> 0.999	> 0.999	> 0.999	55%	2.34 ± 1.07	2.36 ± 1.10	2.59 ± 1.20	> 0.999	**0.014**	0.051
60%	2.81 ± 0.94	2.80 ± 0.94	2.80 ± 0.94	> 0.999	> 0.999	> 0.999	60%	2.38 ± 1.20	2.38 ± 1.20	2.55 ± 1.23	1.000	0.086	0.086
65%	2.83 ± 0.87	2.81 ± 0.85	2.85 ± 0.91	> 0.999	> 0.999	0.625	65%	2.52 ± 1.20	2.500 ± 1.20	2.71 ± 1.16	> 0.999	**0.020**	**0.011**
70%	2.84 ± 0.83	2.86 ± 0.85	2.97 ± 0.82	> 0.999	0.109	0.180	70%	2.60 ± 1.16	2.62 ± 1.19	2.73 ± 1.20	> 0.999	0.094	0.109
75%	3.10 ± 0.84	3.11 ± 0.85	3.19 ± 0.84	> 0.999	0.125	0.141	75%	2.86 ± 1.09	2.80 ± 1.09	2.96 ± 1.08	0.250	0.180	**0.022**
80%	3.05 ± 0.73	3.03 ± 0.75	3.23 ± 0.82	> 0.999	**0.008**	**0.001**	80%	3.02 ± 0.88	2.94 ± 0.85	3.04 ± 0.89	0.125	> 0.9999	0.180
85%	3.00 ± 0.83	2.98 ± 0.85	3.10 ± 0.88	> 0.999	0.125	**0.031**	85%	2.82 ± 0.82	2.80 ± 0.88	2.95 ± 1.01	> 0.999	0.156	0.074
90%	2.86 ± 0.74	2.87 ± 0.78	3.00 ± 0.81	> 0.999	0.063	0.109	90%	2.79 ± 0.84	2.80 ± 0.85	2.88 ± 0.98	> 0.999	0.162	< 0.001
95%	2.71 ± 0.71	2.79 ± 0.74	2.71 ± 0.69	0.125	> 0.999	0.219	95%	2.42 ± 0.95	2.47 ± 0.97	2.55 ± 0.98	0.250	0.124	0.388
all	2.75 ± 0.87	2.75 ± 0.87	2.92 ± 0.90	0.752	< 0.001	< 0.001		2.50 ± 1.03	2.50 ± 1.03	2.7 ± 1.06	0.906	< 0.001	< 0.001

Values are given as mean ± standard deviation. Bold type highlights those with a *P*-value below 0.05, indicating significant difference. A2, middle portion of the anterior mitral leaflet; P2, middle scallop of the posterior mitral leaflet; SSF, SnapShot Freeze 1.0; SSF2, SnapShot Freeze 2.0; STD, Standard

- Subjective image quality scores of the systolic-phase images.

**Fig. 5:**
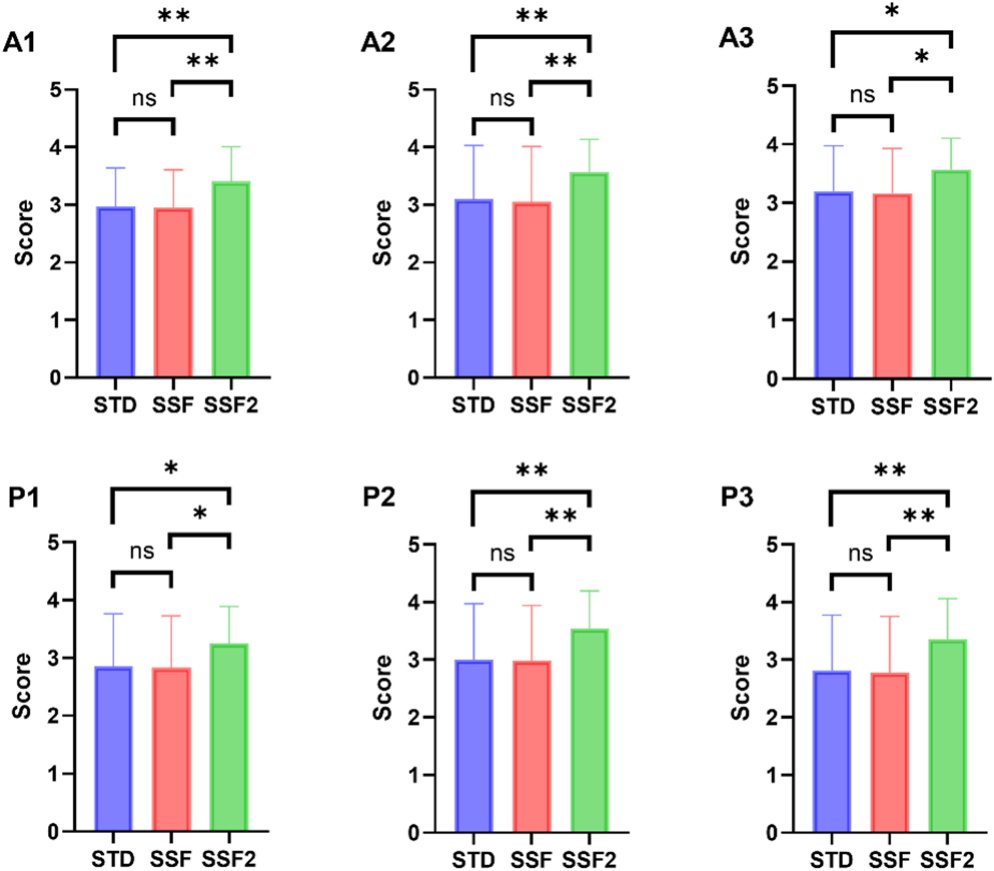
Comparison of image quality scores according to reconstruction method. A1, lateral portion of the anterior mitral leaflet. A2, middle portion of the anterior mitral leaflet. A3, medial portion of the anterior mitral leaflet. ns, not significant. P1, lateral scallop of the posterior mitral leaflet. P2, middle scallop of the posterior mitral leaflet. P3, medial scallop of the posterior mitral leaflet. SSF, SnapShot Freeze 1.0. SSF2, SnapShot Freeze 2.0. STD, Standard. **P < 0.0001. *P < 0.05

The mean score for all valve leaflets in the systolic phase (A1 to P3), which contributes to the diagnosis of mitral regurgitation, was significantly higher for SSF2 (3.46 ± 0.61) than for STD (3.03 ± 0.86) and SSF (3.00 ± 0.85). Image quality scores for each valve apex (A1, A2, A3, P1, P2, and P3) were significantly higher for SSF2 than the STD and SSF scores but did not differ significantly between SSF and STD (Fig. 5).

**Fig. 6:**
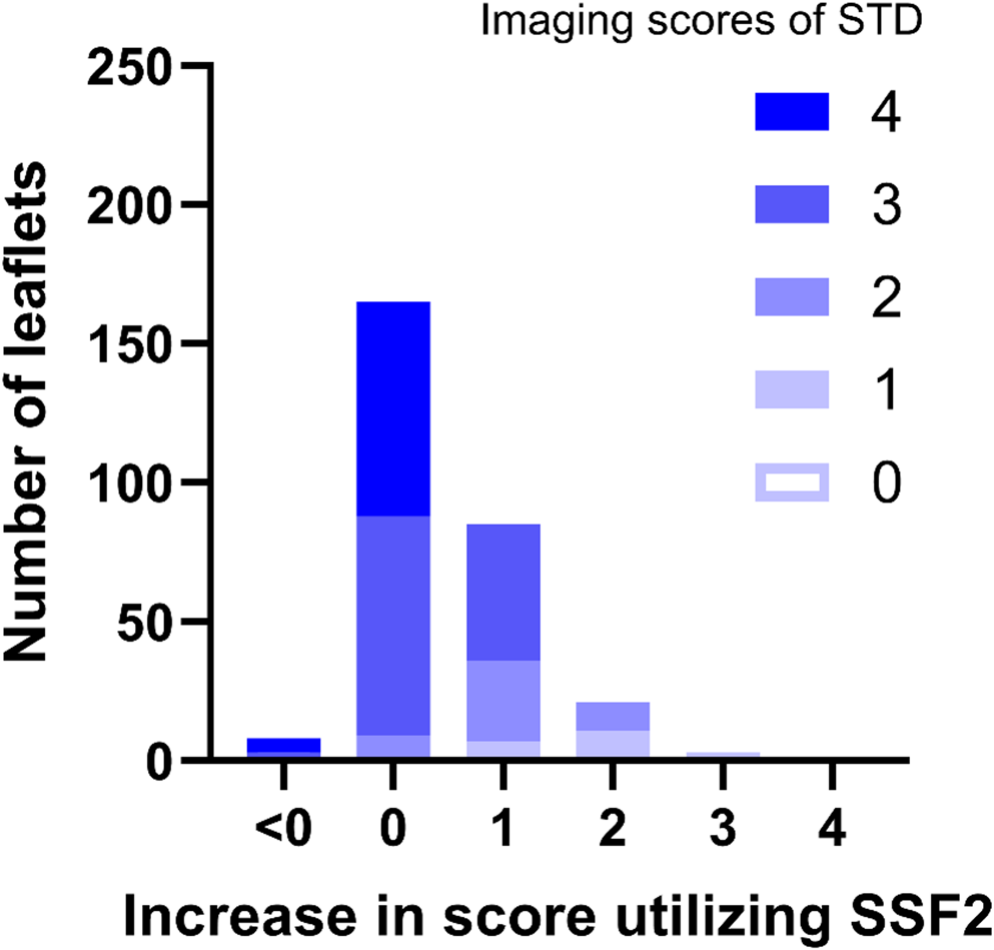
Histogram comparison of standard image quality scores with increase in SnapShot Freeze 2.0 score. SSF2, SnapShot Freeze 2.0. STD, Standard

Table 3 shows the increase in the SSF and SSF2 scores compared with the STD score, i.e., the value obtained by subtracting the STD score from the SSF and SSF2 scores. Compared to STD, scores of five of the total 282 leaflets (1.8%) in SSF and 109 of the 282 (38.7%) in SSF2 improved by one or more. The graphs in Fig. 6 illustrate the breakdown of the score increase in SSF2 compared to STD. The most common score increase, observed in 165 leaflets (58.5%), was below one point, but 79 of these 165 cases had an STD score of three, and 77 had a score of four.

**Table 3:** Increase in the SSF and SSF2 scores compared with the STD score

	SSF	SSF2
< 0	13	8
0 to < 1	264	165
1 to < 2	5	85
2 to < 3	0	21
3 to < 4	0	3
4	0	0

SSF, SnapShot Freeze 1.0; SSF2, SnapShot Freeze 2.0

**Fig. 7:**
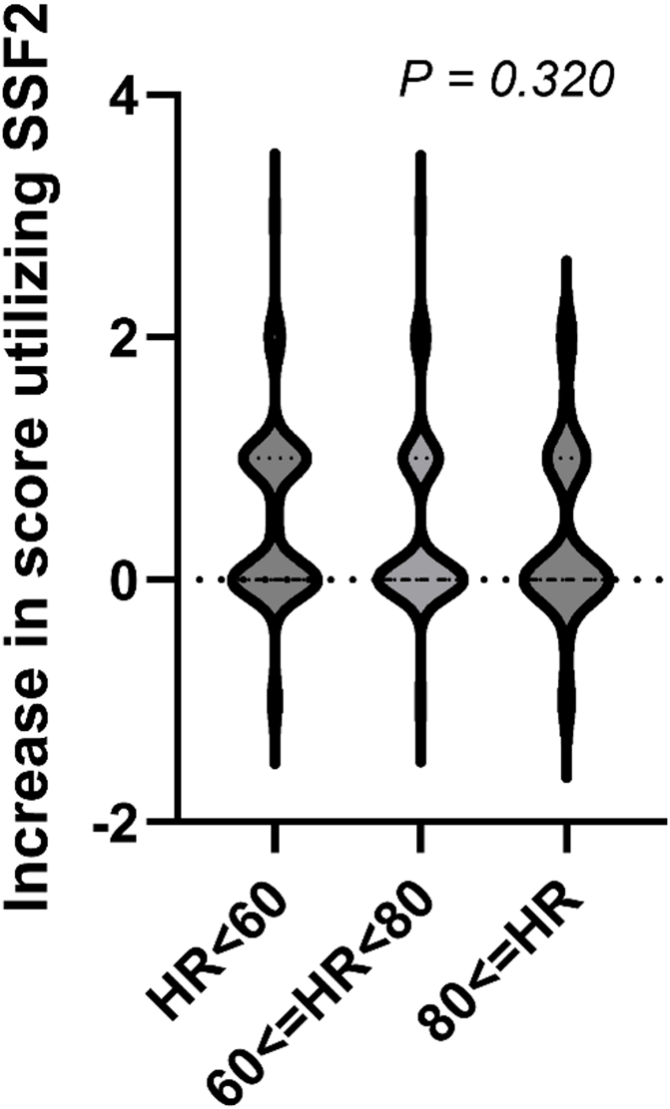
Histogram of score increase distribution based on heart rate. HR, heart rate; SSF2, SnapShot Freeze 2.0. STD, Standard

In both STD and SSF, the mean score for atrial fibrillation (3.20 ± 0.83 in STD; 3.19 ± 0.83 in SSF) was significantly higher than that for sinus rhythm (2.88 ± 0.89 in STD; 2.84 ± 0.89 in SSF). However, in SSF2, the scores for AF (3.48 ± 0.60) and SR (3.44 ± 0.63) did not differ significantly. None of the reconstructions showed significant correlation of heart rate with score during imaging (STD, *P* = 0.886; SSF, *P* = 0.935); and SSF2, *P* = 0.152). When we classified heart rates into three groups (fewer than 60 bpm, 60–80 bpm, and greater than 80 bpm) and examined the increase in scores, score improvement did not differ significantly among the groups based on heart rate (*P* = 0.320) (Fig. 7).

## Discussion

### Subjective image quality scores for all 20 cardiac phases for the A2/P2

In both A2 and P2 images, the average image quality score across all 20 cardiac cycles of the SSF2 technique was significantly higher than those of the STD and SSF techniques. With the exception of the 20% R-R-phase P2 images, the SSF2 scores were significantly higher than those of STD and SSF in the systolic phase from the five to 40% R-R intervals. These results highlight the utility of SSF2 in the systolic phase, which is important for the diagnosis of mitral regurgitation. As with the first-generation algorithm, SSF2 uses information from adjacent cardiac phases available from a single rotation to characterize motion at the prescribed target phase. In a fully automated manner, SSF2 searches each region of the image volume for a local motion path that is consistent with the subset of measurement data that passes through that portion of the image volume [14]. Each image volume in the series is then spatially deformed by the motion field, which maps the motion state from the respective time to the central reference time given by the prescribed cardiac phase. Motion correction of the second-generation technology extends beyond the coronary vessels to the entire heart [14]. In this study, the diastolic phase of the ventricle is the period of the cardiac cycle from the closure of the aortic valve to the closure of the mitral valve during which the ventricle is filled with blood, and SSF2 reconstruction did not improve mitral valve image quality during this time. Diastole comprises four sequential components‒ isovolumic relaxation, early rapid filling, diastasis, and atrial systole (late filling). During the isovolumic relaxation phase, relaxation and lowering of internal pressure of the left ventricle coincide with closure of both the aortic and mitral valves, creating a pressure gradient in which left atrial pressure exceeds left ventricular pressure. Early rapid filling then occurs as blood passively flows from the left atrium to the left ventricle utilizing this pressure gradient. Diastasis (cardiac quiescence) follows as the influx of blood raises left pressure, momentarily pausing flow and equalizing the left ventricular and left atrial pressure, and as the remaining blood is actively propelled into the left ventricle during atrial systole (late filling), diastole is completed. Blood flows from the left atrium to the left ventricle during the nonsequential periods of early rapid and late filling [15], so SSF2 may have been unable to perform motion correction during diastole, when the mitral valve exhibits complex motion, because the algorithm uses images of adjacent cardiac phases to correct for motion in the target cardiac phase.

Table 2 shows lower image quality scores for the posterior leaflet than the anterior leaflet in each reconstruction, presumably attributable to the shorter length of the posterior than anterior leaflet and its close proximity to the left ventricular wall during diastole, which may have hindered its clear recognition.

### Subjective image quality scores of the systolic-phase images

The mean score of image quality for all systolic leaflets (A1-P3), which are important for the diagnosis of mitral regurgitation, was significantly higher in SSF2 (3.46 ± 0.61) than in STD (3.03 ± 0.86) and SSF (3.00 ± 0.85). For individual valve cusps (A1, A2, A3, P1, P2, and P3), image quality did not differ significantly between the SSF and STD reconstructions but was consistently significantly higher for SSF2 than both STD and SSF. Because the first-generation algorithm (SSF) only corrects coronary artery motion [9], the mitral valve image score probably did not improve. However, the second-generation algorithm (SSF2) corrects motion in other structures in the heart in addition to the coronary arteries, so its use likely improved the quality of mitral valve images [9] and therefore assessment of mitral valve regurgitation.

In the SSF group, scores of only 1.8% of cases increased by one or more, whereas in the SSF2 group, scores of 38.7% of cases improved, suggesting that SSF2 reconstruction positively affects mitral valve image quality. Even in the SSF2 group, scores of 61.3% of cases showed no improvement. However, among the SSF2 cases with score increase below one, 94.8% had a pre-motion-correction score (STD) of three or four, indicating an already high initial score.

Sixteen of our 47 cases (34%) demonstrated atrial fibrillation. Though coronary computed tomography angiography (CCTA) represents a more technically challenging application of cardiac CT for the assessment of coronary arteries in patients with AF, other investigators have reported the utility of CT scanners with whole-heart coverage in acquiring high-quality CCTA images while reducing radiation exposure [16, 17]. In our study as well, use of CT scanners with whole-heart coverage produced equivalent or better image quality of the mitral valve in atrial fibrillation than that in sinus rhythm, even without motion correction. Pulsed Doppler echocardiography shows distinct E (early rapid filling velocity) and A (late diastolic filling velocity, specifically reflecting left atrial contraction) waves, representing early and atrial filling of the left ventricle (LV) via the mitral valve (MV) [15]. In AF, the A-wave is particularly absent. Consequently, without motion correction, i.e., in STD, higher AF than SR scores may have resulted from the reduced valve motion associated with the loss of atrial transport (atrial kick) characteristic of AF. Notably, the absence of significant difference between the AF and SR scores in the SSF2 group suggests a potential benefit of the SSF2 motion-correction algorithm.

None of the three reconstructions, STD, SSF, or SSF2, showed significant correlation between heart rate and score during imaging, suggesting the possibility of equivalent image quality in cases of low and high heart rate. Conversely, in the case of low heart rate, the long time between each phase in the 20-phase reconstruction might prohibit capture of the optimal cardiac phase.

### Scanning protocol and analysis

Our standard coronary CTA protocol uses a contrast agent injection rate of 22 mgI/kg/sec. However, in cases of mitral regurgitation, many patients have left atrial enlargement, so we increased the injection rate to 28 mgI/kg/sec. Because it is difficult to obtain cross-sections perpendicular to the anterior and posterior leaflets at the A1/P1 and A3/P3 during diastole, we evaluated only the systolic phase, which contributes to diagnosis.

ECG-gated CT prior to TAVI has been reported to enable the evaluation of both the aortic valve and coronary arteries with reconstruction at 20 to 30% of the R-R interval [9]. The diagnosis of mitral regurgitation requires images obtained during the left ventricular systole, when the mitral valve is closed, and among left ventricular systolic phases ranging from 15 to 40% of the R-R interval, we observed no significant difference in heart rate during the scan among the best systolic phases of the images. So, modulating contrast dose at five to 40% of the R-R interval may reduce radiation exposure. However, comprehensive evaluation is essential prior to mitral valve plasty, percutaneous mitral annuloplasty, and edge-to-edge mitral valve repair for mitral regurgitation. Beyond CT assessment of leaflet morphology for prolapse or billowing, accurate characterization of the coronary arteries, mitral annulus, left ventricular geometry, including the chordae tendineae and papillary muscles, and the subvalvular apparatus is mandatory [5, 18, 19]. As well, evaluating deformation of the mitral annulus is crucial for procedures aimed at restoring MV geometry. Thus, both the left ventricular diastolic and systolic phases are considered to play important roles. In addition, the many cases of atrial fibrillation make it difficult to predict the heart rate at the time of imaging, thereby requiring the acquisition of images in both the diastolic and systolic phases. In CT for preoperative evaluation of mitral regurgitation, the implementation of dose modulation to reduce radiation exposure will very likely remain difficult in the future. To cope with sudden heart rate fluctuations, we also set the R-R intervals to 120% or more and performed imaging of the left heart and coronary arteries.

### Limitations

Our retrospective study was limited because we included only 47 patients with mitral regurgitation, which did not provide sufficient data to allow evaluation of the impact of this reconstruction method on the differential diagnosis of functional or degenerative disease. In addition, we divided the R-R interval of the electrocardiogram into 20 parts and evaluated the quality of mitral valve images, but we may have failed to capture the optimal cardiac phase in cases of low heart rate.

Furthermore, though comprehensive evaluation of visibility requires objective quantitative analyses, specifically the use of CT value profiling for leaflet thickness and direct measurement of leaflet length, significant technical and inherent limitations in the acquired data restricted our study to the subjective, qualitative assessment of mitral valve leaflet visualization by two radiologists.

The limitations of CT profile curve analysis of the mitral valve are primarily attributable to motion artifacts and the inherent thinness of the leaflets. Because CT values are represented on a per-pixel basis, the attenuation values of the thin leaflets are highly susceptible to image noise and partial volume effect. Consequently, the CT value at the nadir of the leaflet, which exhibits lower attenuation than the intracardiac lumen, increases, thus reducing the accuracy of FWHM measurements or rendering those measurements impossible by preventing detection of the leaflet. FWHM measurement is further challenged by the method of image reconstruction. The inherent requirement of techniques such as SSF and SSF2 to utilize cardiac phases preceding and subsequent to the target phase prohibits the perfect coregistration of coordinate axes of the three distinct image datasets produced by differing reconstruction methods. This lack of precise alignment makes it difficult to measure FWHM at the exact same anatomical location across all reconstructed images.

Objective measurement of leaflet length was difficult because motion blur prevented clear depiction of the leaflets, and indistinct delineation hindered the accurate determination of their boundaries. As well, overlap of the anterior and posterior leaflets during coaptation prevents measurement to the true tip of the leaflet.

Because this study is exploratory in nature, we did not perform corrections for multiple comparisons, so our findings should be interpreted with caution and used primarily for hypothesis generation for future confirmatory studies.

## Conclusion

In conclusion, SSF2 reconstruction effectively mitigates motion artifacts to provide mitral valve images of superior quality in preoperative CT for mitral regurgitation. Reflected across different cardiac phases and independent of heart rhythm and rate, our findings suggest the robustness of SSF2 for use in various clinical settings and to aid surgical planning.

## Data Availability

The datasets used and analyzed during the current study are available from the corresponding author on reasonable request.

## Abbreviations

A1: Lateral portion of the anterior mitral leaflet
A2: Middle portion of the anterior mitral leaflet
A3: Medial portion of the anterior mitral leaflet
bpm: Beats per minute
CCTA: Coronary computed tomography angiography
CT: Computed tomography
CTA: Computed tomography angiography
CTDIvol: Computed tomography dose index volume
ΔHU: Delta Hounsfield unit
DLP: Dose length product
ECG: Electrocardiography
ED: Effective dose
eGFR: Estimated glomerular filtration rate
FWHM: Full width at half medium
HR: Heart rate
IVSd: Interventricular septum diameter
LAV: Left atrial volume
LAVI: Left atrial volume index
LVDd: Left ventricular diastolic diameter
LVDs: Left ventricular systolic diameter
LVEF: Left ventricular ejection fraction
MRF: Mitral regurgitation fraction
MRV: Mitral regurgitation volume
ns: Not significant
P1: Lateral scallop of the posterior mitral leaflet
P2: Middle scallop of the posterior mitral leaflet
P3: Medial scallop of the posterior mitral leaflet
PWd: Posterior wall diameter
R-R: R-peak to R-peak
ROI: Region of interest
SNR: Signal-to-noise ratio
SSF: SnapShot Freeze 1.0 motion-correction algorithm
SSF2: SnapShot Freeze 2.0 motion-correction algorithm
STD: Standard reconstruction of images without motion correction
